# Cultural adoption, and validation of the Persian version of the coronary artery disease education questionnaire (CADE-Q): a second-order confirmatory factor analysis

**DOI:** 10.1186/s12872-020-01628-5

**Published:** 2020-07-23

**Authors:** Zahra Marofi, Razieh Bandari, Majideh Heravi-Karimooi, Nahid Rejeh, Ali Montazeri

**Affiliations:** 1 Faculty of Nursing & Midwifery, Shahed University, Tehran, Iran; 2 Social Determinants of Health Research Center, Semnan University of Medical Sciences, Semnan, Iran; 3 Elderly Care Research Center, Faculty of Nursing & Midwifery, Shahed University, Tehran, Iran; 4 Health Metrics Research Center, Iranian Institute for Health Sciences Research, ACECR, Tehran, Iran; 5 Faculty of Humanity Sciences, University of Science & Culture, ACECR, Tehran, Iran

**Keywords:** Coronary artery disease, Psychometric analysis, Reliability, Validity, Knowledge, The CADE-Q

## Abstract

**Background:**

Evaluating knowledge in patients with coronary artery disease requires a specific measure. The aim of the present study was to translate and evaluate the CADE-Q in patients with coronary artery disease in Iran.

**Methods:**

Forward-backward procedure was applied to translate the questionnaire from English into Persian. Then a cross-sectional study was conducted to evaluate psychometric properties of the questionnaire. A sample of patients with coronary artery disease attending to cardiac departments of teaching hospitals affiliated to medical universities in Tehran, Iran completed the 19-item CADE-Q from April to December 2017. Structural validity of CADE-Q was assessed using both exploratory and confirmatory factor analyses. Reliability was examined using Cronbach’s alpha coefficient. Stability was evaluated by estimation intraclass correlation coefficient.

**Results:**

In all 500 patients participated in the study. The mean age of patients was 53.63. (SD = 14.36) years, and 57% were male. The results obtained from the exploratory factor analysis showed a four factor solution (lifestyle habits and exercise, risk factors, diagnosis and treatment, signals & symptoms and medicine) that jointly explained 48.9% of the total variance observed. However, the second-order confirmatory factor analysis supported the three-factor solution while convergent and divergent validity were not confirmed. Finally, the Cronbach’s alpha coefficient of 0.84 ranging from 0.50 to 0.82 was obtained for the scale and its subscales. In addition, the ICC value of 0.88 showed satisfactory stability for the questionnaire.

**Conclusion:**

The Coronary Artery Disease Education Questionnaire was found to be a multidimensional instrument. The results confirmed the factor structure of the questionnaire with a second-order analysis. Since the convergent and divergent validity of the scale were not confirmed, further assessment is essential to establish fitness of the questionnaire in Iran.

## Background

Cardiovascular diseases account for around 16.7 million deaths per year worldwide and it has been estimated that the figure will increase to 23.6 million cases by 2030 [1–3]. In order to reduce mortality among patients who suffer from cardiac diseases there are many strategies. Of these, cardiac rehabilitation programs are among well-known strategies. The cardiac rehabilitation programs consist of a set of interventions ranging from physical activity, exercise training, post-operative patient care, the optimization of medical treatment, nutritional counseling, smoking cessation, risk stratification, hypertension management, control of diabetes or dyslipidemia, patient assessment, weight management, aggressive coronary risk factor management, psychosocial counseling, sexual dysfunction, alcohol consumption and stress management [2, 4, 5]. However at the heart of all these programs education is an important component [6–8].

Studies have shown that rehabilitation programs have significant effect in reducing length of hospital stay, improving the quality of life, and patient’s performance [9]. In fact such programs could raise patients’ level of knowledge and help him/her in making healthy choices in everyday life [10]. Patient education is a process that affects the patients’ behavior and changes their knowledge, attitudes, and skills needed to maintain health status [11].

Considering the paramount importance of knowledge in patients with coronary artery disease, various tools have been used to measure their level of knowledge. These tools include the MICRO-Q (Maugeri Cardiac Prevention Questionnaire) [12], the Cardiac Rehabilitation Barriers Scale [13, 14], the Information Need in Cardiac Rehabilitation Scale [15], the Knowledge Questionnaire for Cardiac Failure Patient [16], original version of the Coronary Artery Disease Education Questionnaire, CADE-Q [17], the second version of the CADE-QII [18], and short version of the CADE-Q [19].

Although all this questionnaire are valid instruments, the English version of the CADE-Q seems perform better since it is shorter compared to the other questionnaires and uses four-response categories while most existing questionnaire use true/falls format. As such as part of a project on raising knowledge among patients with coronary artery disease the aim of this study was to translate and validate the English version of the CADE-Q in Iran. Currently no such a questionnaire is available in Iran we aimed.

## Methods

### Study design and sample

This was a cross sectional study on a sample of patients with coronary artery disease. All patients approached before surgery and the main investigator (ZM) collected the data. All participants were asked to complete the study questionnaires in a calm setting. In the case of illiterate individuals, the main investigator helped people to complete the questionnaires. In all instances completion of the questionnaires took about 15 min. The inclusion criteria were as follows: suffering from CAD, minimum age of 18 years; Iranian; and willing to participate in the study and not suffering from hearing loss or any mental or cognitive disorders. This latter condition was examined by a self-report questionnaire. Patients with other disease conditions were not included. In addition all patients were asked to respond to a short demographic questionnaire including items on age, martial status, education, employment, income, smoking, and history of hospitalization. In Particular income was assessed based on the following single item: overall how would you describe your current income condition. The response category included as poor, intermediate, and good. This was based on similar studies conducted in Iran lending support to its validity [20].

### Sample size justification

For the study purposes we thought 200 patients with coronary artery disease (10 participants per item) are needed for exploratory factor analysis (EFA) and 250 patients are needed for confirmatory factor analysis (CFA) [21, 22]. However, in practice overall we recruited 500 patients with coronary artery disease who were hospitalized in cardiac departments of hospitals affiliated to medical schools in Tehran, Iran.

### The coronary artery disease education questionnaire (CADE-Q)

The Coronary Artery Disease Education Questionnaire (CADE-Q) evaluates patients’ knowledge about their disease and related factors. The questionnaire was developed following a general review of the literature, with feedback from cardiologists and a cardiac rehabilitation multidisciplinary team. The CADE-Q initially validated in Brazilian Portuguese and was confirmed to be a valid tool, with strong overall characteristics in terms of content, development and testing [13]. Then an English version of the questionnaire was developed including 19 items covering five areas of knowledge: pathophysiology, signals and symptoms of the disease; risk factors, prevention of risk factors (lifestyle habits); diagnosis, treatment and medicines; and exercise [Additional file 1]. Each item has four statements that according to knowledge levels, the following criteria will apply: most correct answers = 3; somewhat correct answers = 1; wrong answer = 0; and don’t know = 0. The sum of scores could be computed to calculate the knowledge score [23].

### Translation procedure

Forward-backward translation procedure was used to translate the English version of the questionnaire into Persian. As such, two independent professionals translated the questionnaire from English into Persian. Then a consolidated Persian version of the two translations was provided with the best translation available. Subsequently two experts back translated the Persian version into English and it was compared with the original English version by the research team and the provisional version of the Persian version was provided. In order to examine content validity, 10 experts (two cardiologists, five assistant professors in nursing, and three assistant professors experienced in questionnaire design) were asked to qualitatively examine the questionnaire, and provide their opinions on the questionnaire in terms of grammar, vocabulary, necessity, importance, placement of the words, and scoring. The experts made no changes to the questionnaire. Then, the CADE-Q was administered to 10 patients with coronary artery disease who met the inclusion criteria with maximum variance in order to assess the face validity of the questionnaire. Their views on appropriateness, difficulty, relevancy and ambiguity of the items were assessed. Almost all patients did not indicate any problems and thus the questionnaire was made ready for psychometric evaluation.

### Statistical analysis

The following analyses were performed in order to assess the psychometric properties of the questionnaire:

1. Item analysis

Inter-item correlation was assessed in order to suggest if there was any potentially problematic item. It was expected that all correlations range between 0.3 and 0.7 [24].

2. Structural validity
  (i) The exploratory factor analysis (EFA) was performed to extract latent factors. The Kaiser-Meyer-Olkin (KMO) test for sampling adequacy and the Bartlett’s test for sphericity were used. The KMO values between 0.7 and 0.8 are consider to be as good, and values between 0.8 and 0.9 are consider as excellent [25]. Then, the latent factors were extracted using the maximum likelihood estimation, the promax rotation, and scree plots. Presence of each item in the factor was determined according to communalities of above 0.3 in the EFA [26].
  (ii) In the second step, the factors extracted using the first- and second-order factor analysis and the most common goodness of-fit indices of the proposed model were assessed based on the threshold of acceptance and the maximum likelihood approach. There are no golden rules for evaluating goodness-of-fit indices; however, it is necessary to report a variety of indices because different indices often reflect different features of the model [27]. Fit indices employed in the study included Chi-square (χ2), Chi-square/degree-of-freedom ratio (normalized Chi-square CMIN/DF), Adjusted Goodness-of-Fit Index (AGFI), Parsimonious Comparative Fit Index (PCFI), Comparative Fit Index (CFI), Incremental Fit Index (IFI), Parsimonious Normed Fit Index (PNFI), Root Mean Square Error of Approximation (RMSEA) [28]. In the second-order factor analysis, it was assumed that the extracted latent variables in the first stage were present. Thus, the second-order factor analysis represented the more general concepts at secondary and upper levels [29].
  (iii) Convergent and divergent validity were assessed using the average variance extracted (AVE), the maximum shared squared variance (MSV), and the average shared squared variance (ASV) The convergent validity to be stablished, the AVE should be above 0.5, and in order for the divergent validity to be stablished, the ASV and the MSV should be lower than the AVE [30].
  (iv) Sensitivity was examined by extracting factor loadings for patients with or without a history of the hospitalization. It was expected to achieve similar factors for both groups .
3. Reliability assessment
  (i) In order to assess the internal consistency of the CADE-Q, the Cronbach’s alpha coefficient was estimated first for the whole questionnaire and then for each extracted factor. An alpha value above 0.7 was considered to indicate good internal consistency [31].
  (ii) Stability was assessed using the intraclass correlation coefficient (ICC). When this index is above 0.75, there is a good level of stability [32].
4. Normal distribution of data

The normal distribution of the data, the outliers, and missing data were separately assessed. The presence of multivariate outliers was assessed using the Mahalanobis d-squared method (*p* < 0.001) and the violation of multivariate kurtosis using the Mardia coefficient (> 8) [33] The number of missing data was assessed using multiple imputation analysis, which was then replaced with participants’ mean responses. Data analysis was carried out using SPSS 16.0 (SPSS Inc., Chicago, Illinois) and AMOS 17.0 (SPSS Inc., Chicago, Illinois) statistical packages.

## Results

### Patients’ characteristics

In all 500 patients with coronary artery disease were approached and all agreed to took part in the study. Of these 285 (57%) were male, 73.8.0% (*n* = 369) married, and the mean age of participant was 53.6 (SD = 14.36) years, and indicated themselves as having intermediate economic status (58.4%). Most participants reported that they had been hospitalized at least once in the last year (65.8. %). The characteristics of the participants are shown in Table 1.

**Table 1:** Demographic Characteristics of the study sample (*n* = 500)

Gender	Number	%
Male	285	57
Female	215	43
**Age group (years)**		
21–40	97	19.4
41–60	246	49.2
61–80	139	27.8
> 80	18	3.6
**Marital status**		
Married	369	73.8
Single	46	9.2
Widow	74	14.8
Divorced	11	2.2
**Employment status**		
Housewife	184	36.8
Employed	173	34.6
Unemployed	45	9
Retired	98	19.6
**Educational level**		
Illiterate	114	22.8
Primary	119	23.8
Secondary	181	36.2
Higher	86	17.2
**History of smoking**		
Yes	83	16.6
No	417	83.4
**Income**		
Poor	123	24.6
Intermediate	292	58.4
Good	85	17
**Hospitalization history (**in the last year**)**		
Yes	329	65.8
No	171	34.2

### Item analysis

The inter-item correlation was estimated among items and a satisfactory results obtained. The results showed a positive and significant correlation between all items [Additional file 2].

### Exploratory factor analysis

The sampling adequacy (KMO) was calculated as 0.842 and Bartlett’s test was calculated as (*p* < 0.001). The EFA resulted in the extraction of four factors, which explained 48.9% of the total variance (Table 2).

**Table 2:** The component matrix for items and total variance observed resulted from maximum likelihood analysis after promax rotation for the CADE-Q (*n* = 250)

Factor	Factor loading
**Lifestyle habits & exercise**	
19-Which interventions can extend and improve a patient’s quality of life for persons recovering from a cardiac event?	0.95
15-Which of the following changes in the body resulting from regular physical exercise are most important to long term cardiac health?	0.67
18-Which of the statements below regarding psychological stress is most correct?	0.62
14-Guidelines for Physical Activity for people with coronary disease should be based upon which of the following:	0.58
13-Based on your knowledge about exercise and CAD, choose the most appropriate statement below:	0.55
4- Which of the following statements is most accurate regarding our understanding of CAD?	0.49
16-Which of the following statements best describes the pattern for exercise activity in persons recovering from a heart event:	0.47
* Eigenevalue*	*4.32*
* % variance*	*26.35*
**Risk factors**	
3-Which description below is a typical symptom of CAD	0.47
2-Which factors have the most influence on the risk of myocardial infarction.	0.45
6-Of the investigations listed below, which ones provide the most precise information about the diagnosis and prognosis of CAD?	0.36
5-The best time of the day for people with coronary disease to carry out their prescribed exercise is:	0.31
7-Which of the following statements about the management of blood cholesterol levels is most accurate?	0.30
*Eigenevalue*	*1.95*
*% variance*	*4.92*
**Diagnosis and treatment**	
1-Coronary Artery Disease (CAD) is:	0.75
17-Which of the following statements is the most appropriate guidance around levels of blood pressure levels in persons with CAD:	0.53
10-Which values for LDL cholesterol and HDL cholesterol are the optimal targets persons with established CAD (values in mmol/litre)?	0.39
11-In which of the following situations would you avoid carrying out your regular physical exercise?	0.36
*Eigenevalue*	*3.18*
*% variance*	*2.67*
**Signals & symptoms and medicine**	
12-While walking, if you experience a new episode of severe chest discomfort that you think that is angina, you should:	0.38
8-Which of the following statements about the use of “nitroglycerin” is most accurate	0.30
*Eigenevalue*	*2.24*
*% variance*	*2.15*

### Confirmatory factor analysis

**Fig. 1:**
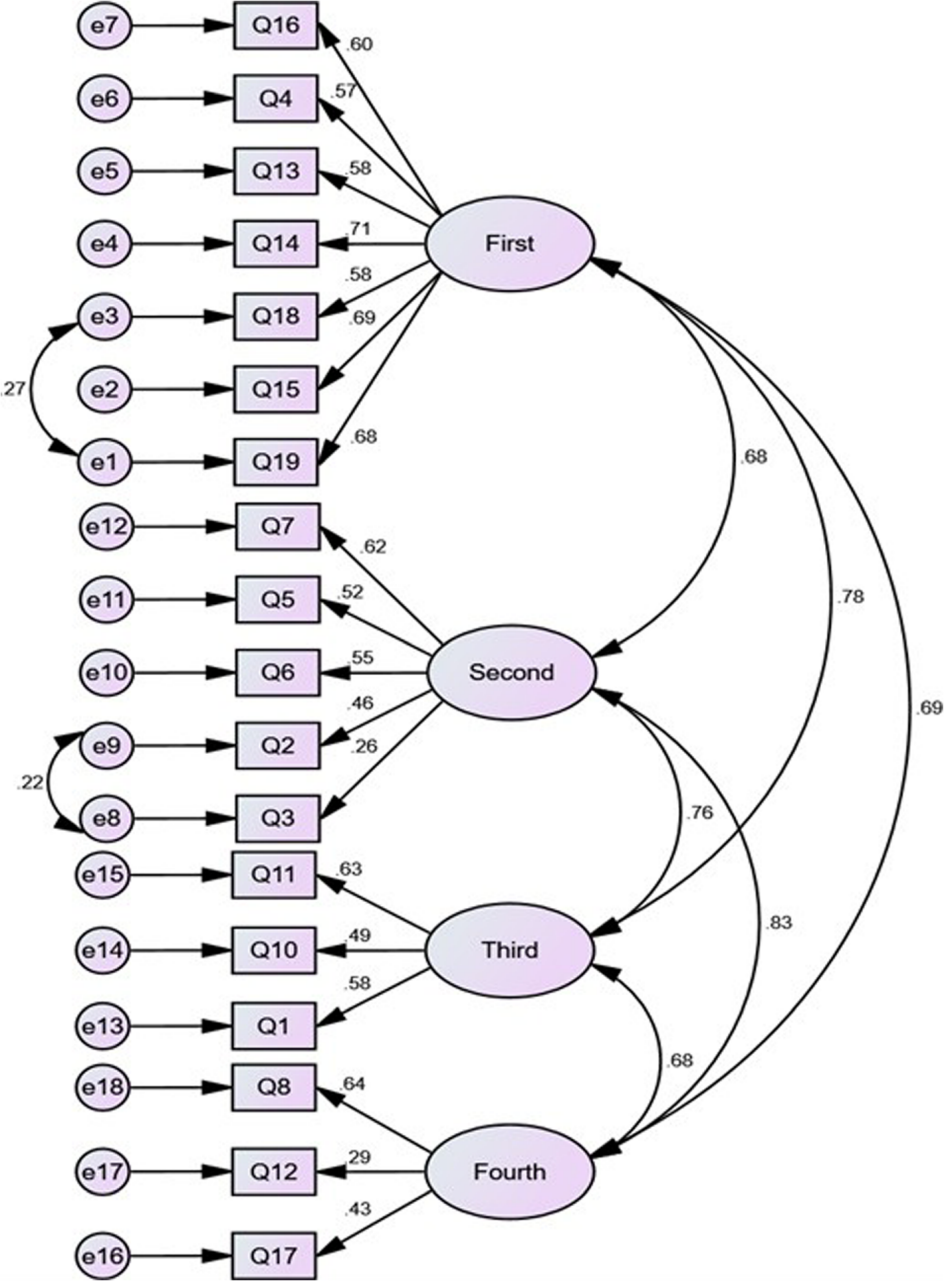
First-order confirmatory factor analysis

The results of the Chi-square test for goodness-of-fit were first obtained and other indices were then assessed for the fit of the model. According to Table 3, all the indices confirmed a good fit of the final model. According to the final factor structure of the CADE-Q, correlations between the measurement errors of items 2 and 3, 18 and 19 were observed (Fig. 1).

**Table 3:** The range of acceptable fit indexes for confirmatory factor analysis

Indexes*	Cut off values	First order	Second order
CMIN/df	< 3	2.08	2.08
*P* value**	≥0.05	0.0001	0.0001
GFI	≥0.90	0.96	0.96
AGFI	≥0.90	0.94	0.94
RFI	≥0.90	0.91	0.91
TLI	≥0.95	0.94	0.94
IFI	≥0.90	0.96	0.96
CFI	≥0.95	0.96	0.96
PCFI	≥0.5	0.70	0.70
PNFI	≥0.5	0.70	0.73
RMSEA	≤0.08	0.04	0.04

* *Abbriviations*: *CMIN/DF* Minimum Discrepanscy Funcation by degrees of freedom divided, *GFI* Good of Fit Index, *AGFI* Adjusted Good of Fit Index, *RFI* Relative Fit Index, *TLI* Tucker –Lewis Index, *IFI* Incremental Fit Index, *CFI* Comparative Fit Index, *PCFI* Parsimonious Comparative Fit Index, *PNFI* Parsimonious Normed Fit Index, *RMSEA* Root Mean Square Error of Approximation

** Derived from chi-square test (χ^2^), which is one of measures for fit indexes in structural equation modeling (SEM). A non-significant result for this test indicates good model fit (i.e., *p* > .05)

**Fig. 2:**
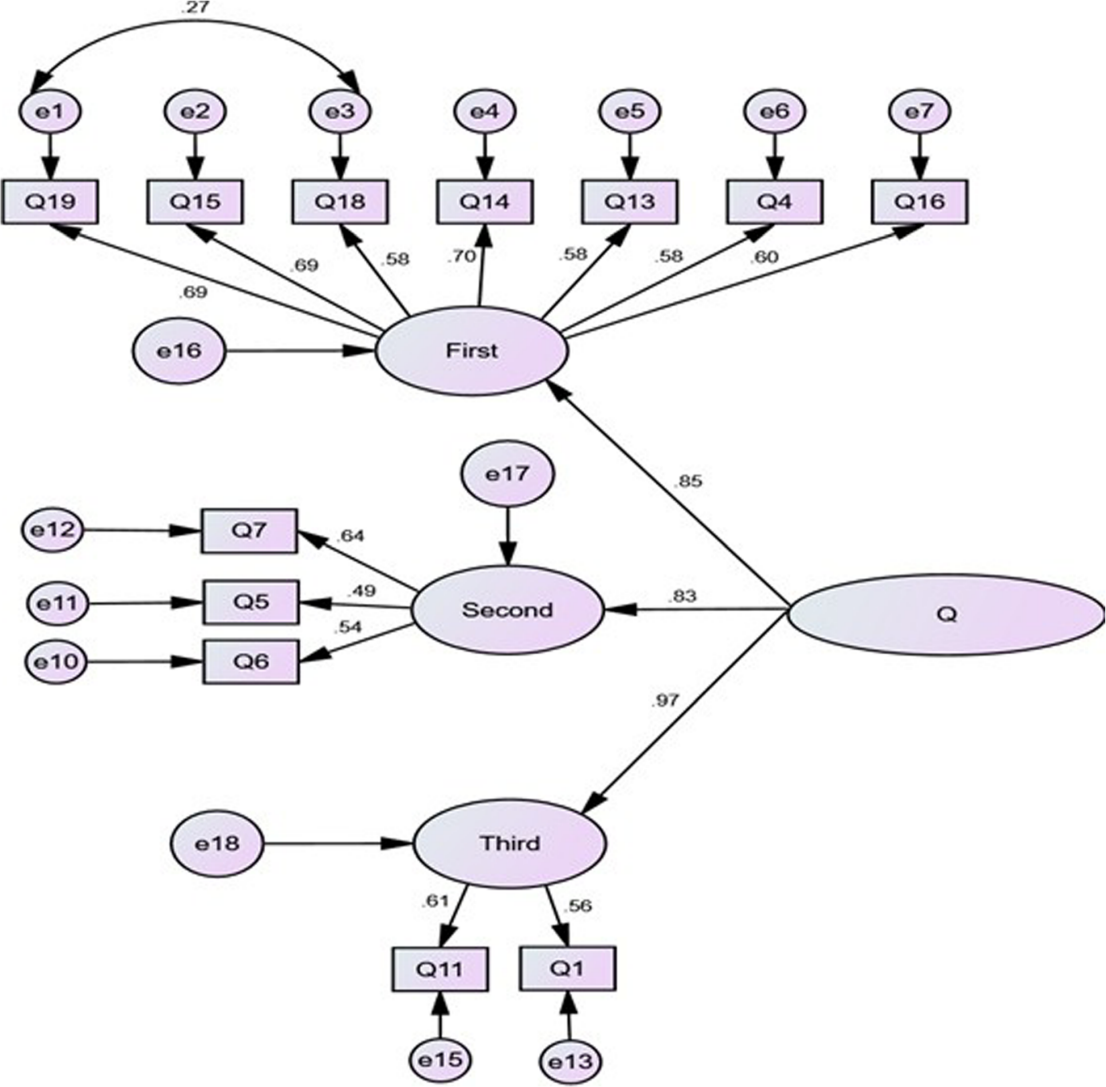
Second-order confirmatory factor analysis

Following the first-order CFA, a separate assessment of the factors of the CADE-Q and the correlation between the constructs occurred. Subscales were determined using structural equations. The second-order factor analysis was performed to examine whether or not all the factors fitted the general concept of “knowledge.” Table 3 presents the indices of fit for the second-order CFA compared to the first-order model. Figure 2 shows the structural model and the CFA of the CADE-Q with standardized factor loadings. The factor loadings were greater than 0.3 for all the items and were significant at *p* < 0.05.

### Convergent and discriminant validity

Assessing the AVE (0.40, 0.31, 0.34), MSV (0.67, 0.64, 0.67) and ASV (0.56, 0.58, 0.66), however, showed that the CADE-Q does not have a good convergent and divergent validity.

### Sensitivity analysis

Sensitivity as assessed by exploratory factor analysis for patients with or without history of the hospitalization indicated similar factor extraction as expected [Additional file 3].

### Reliability

Finally, a Cronbach’s alpha of 0.84 ranging from 0.50 to 0.82 was found for the whole scale and the subscales, respectively. In addition, an ICC of 0.88 ranging from 0.78 to 0.87 was found for the whole scale and the subscales, respectively (Table 4).

**Table 4:** The Cronbach’s alpha and the Intraclass Correlation Coefficients (ICC) for the Persian version of the CADE-Q

Dimension	Cronbach’s α	ICC (***n*** = 100)
**Lifestyle habits & exercise**	0.825	0.870
**Risk factors**	0.553	0.782
**Diagnosis and treatment**	0.507	0.825
**Total score**	0.844	0.886

## Discussion

The present study was aimed to translate the Coronary Artery Disease Education Questionnaire (CADE-Q) into Persian and assess its validity and reliability to be used in epidemiological and clinical studies. We used rigorous methods based on both psychometric and conceptual criteria. The final CADE-Q was shorter than original one with improved fit indices relative to the long version. However, we kept the English version consistent with the original conceptual model.. It covers the key dimensions of the ‘lifestyle habits & exercise’, ‘risk factors’ and ‘diagnosis and treatment’. In addition, reliability, in terms of internal consistency, was preserved in the Persian version. However, one should note that we reduced five dimensions to three dimensions that, to some extent, is not unusual. To explain the issue further it is necessary to acknowledge that there are three CADE-Q versions: one consisting of five dimensions with 19 items (CADE-Q) [23], and the second version containing four dimensions with 31 items (CADE-QII)(18) and the third that is the short version containing four dimensions with 20 items (CADE-Q SV) [19]. Although there were a good similarity between the Persian version and the English version, the difference on number of dimensions might relate to difference in culture and environment. Further investigations might shed more light on this issue.

The first factor identified in the CADEQ includes a general viewpoint of lifestyle and exercise. The exercise concept is broadly about how a person measures the concept of having physical activity in different aspects of his or her life. This factor suggests that the first step in maintaining heart health is to establish a good relationship with health care providers. However, making permanent changes requires learning a series of things, which include heart disease and the types of behaviors and conditions that can increase the risk in a person [34].

The results of EFA showed that the second dimension of CADEQ questionnaire relates the risk factors. Therefore, the best solution includes timely investigation, diagnosis, control of risk factors, and prevention of cardiovascular disease. Making healthy lifestyle changes based on guidelines obtained from decades of research not only prevents cardiovascular diseases, but also reduces the risk of dangerous diseases such as cancer and diabetes [35].

The third factor extracted in the CADEQ questionnaire includes the diagnosis and treatment dimension. Physicians believe that diagnostic tools and methods are very important to evaluate cardiac function for the diagnosis, treatment and follow-up, and have a significant effect on life expectancy and improving the quality of life of patients with coronary artery disease [36].

The fourth factor extracted in the CADEQ relates to the signals & symptoms and medicine. It is important to note that the signals and symptoms of the disease are the most important diagnostic tools in all diseases, including heart disease, and the physician uses diagnostic methods to confirm and prove clinical findings, or complete the required information based on the patient’s history and careful examinations. Therefore, patients with coronary artery disease should be fully aware of this important issue [37].

To find a more precise model of structural equations, the second-order CFA was also performed. This method seeks to obtain a more significant method of data collection while assuming that the latent variables in the common variance are due to one or more higher-order factors and that the intended scale has two orders [29]. A high correlation between the first-order constructs shows that the latent variables do not fully act as an independent variable and the correlation between them reflects the presence of a more general construct (knowledge) in a secondary conceptual level, where the best approach to the assessment of the structure is structural equation modeling, since it can identify the first-order constructs that were proposed as the latent variables [30] Anderson and Gerbing proposed that the intended construct must first be created through first-order factor analysis, and the good fit of the conceptual construct be then determined for the assessment of the structural equation model using second-order factor analysis [38].

Another finding of the present research showed that this scale has a good internal consistency. In this research, the Cronbach’s alpha coefficients of the questionnaire were in the range of 0.577–0.825 and the total Cronbach’s alpha coefficients of the questionnaire was 844; however, the Cronbach’s alpha coefficients was reported to be 0.76–0.94 in Gisis’s study that may be due to the fact that no question has been removed from the above questionnaire in this study [19]. It is worth noting that the Cronbach’s alpha coefficients was expressed in the range of 0.66–0.71 in the second version of the CADEQ questionnaire, which is relatively similar to our study [39]. The reliability of the questionnaire was also evaluated using a test-retest method. The results showed a good stability for the aforementioned questionnaire. The ICC value is between 0.882 and 0.886.

### Limitations

This study had a number of limitations, including the use of the self-report method of data collection, which can entail errors in reporting. Also, since the study was conducted in a certain geographical region, generalizing the results to wider geographical areas should be pursued with care. Future research should consider in different geographical regions.

## Conclusion

Overall, the findings of this study suggest that CADEQ is acceptable in terms of psychometric properties. The questionnaire is easy to use and can be completed both by a patient or healthcare provider in a variety of settings.

## Supplementary information

**Additional file 1.** The Coronary Artery Disease Education Questionnaire (CADE-Q).

**Additional file 2.** Inter-item correlations for the CADE-Q.

**Additional file 3.** The EFA for the CADE-Q among patients with and without hospitalization history.

## Data Availability

The datasets are available from the corresponding authors on request.

## Abbreviations

AGFI: Adjusted goodness of fit index
AVE: Average variance extracted
ASV: Average shared variance
CFA: Confirmatory factor analysis
CFI: Comparative fit index
CMIN/DF: Minimum Discrepanscy Funcation by degrees of freedom divided
EFA: Exploratory factor analysis
GFI: Goodness of fit index
ICC: Intraclass correlation coefficients
IFI: Incremental fit index
KMO: Kaiser–Meyer–Olkin
MSV: Maximum variance
PCFI: Parsimonious comparative fit index,
PNFI: Parsimonious normed fit index
RMSEA: Root mean square error of approximation
SEM: Structural equation modeling
SRMR: Standardized root mean square residual
TLI: Tucker –Lewis index
