# Supplementary material for: Cultural adoption, and validation of the Persian version of the coronary artery disease education questionnaire (CADE-Q): a second-order confirmatory factor analysis
Source: BMC Cardiovasc Disord. 2020 Jul 23;20:345. doi: 10.1186/s12872-020-01628-5 (PMC7379361; doi:10.1186/s12872-020-01628-5)
Supplement: Supplementary file 1 — Additional file 1. The Coronary Artery Disease Education Questionnaire (CADE-Q). [file 12872_2020_1628_MOESM1_ESM.doc]

**The Coronary Artery Disease Education Questionnaire (CADE-Q)**

| **Question** | **The answers** |
| --- | --- |
| 1-Coronary Artery Disease (CAD) is: |  |
|  | a) A disease of the arteries of the heart which occurs in older age in people with high cholesterol and who smoke. |
|  | b) A disease of the arteries of the heart that starts silently at a young age, is influenced by poor life style habits, has a genetic component, and involves inflammation in the arteries. |
|  | c) A disease of the heart’s arteries related to older age and that leads to memory Impairment. |
|  | d) I don’t know. |
| 2-Which factors have the most influence on the risk of myocardial infarction? |  |
|  | a) Drinking small amounts of alcoholic beverages. |
|  | b) Environment factors (such as weather) and socioeconomic factors (such as monthly family income). |
|  | c) Smoking, high levels of blood cholesterol (dyslipidemia), and hypertension. |
| 3-Which description below is a typical symptom of CAD? |  |
|  | a) Headache after meals. |
|  | b) Chest pain or discomfort during physical activity. |
|  | c) Chest pain or discomfort, at rest or during physical activity, which can also be felt in the arm and/or back and/or neck. |
| 4- Which of the following statements is most accurate regarding our understanding of CAD? |  |
|  | a) The CAD is related to blockage of the arteries that supply blood to the heart caused by the formation of atherosclerotic plaques (fat deposit on the arteries’walls) that can cause angina (chest pain). |
|  | b) The Acute Myocardial Infarction (MI) is the only manifestation of CAD. |
|  | c) The presence of chest pain is suggestive of a diagnosis of CAD. |
| 5-The best time of the day for people with coronary disease to carry out their prescribed exercise is |  |
|  | a) In the afternoon or evening, because the early morning is the time of day with the highest risk of a heart attack. |
|  | b) Never, because exercise is considered too risky for people with CAD |
|  | c) Any time, because the benefits of exercise outweigh the risks at any time of day |
| 6-Of the investigations listed below, which ones provide the most precise information about the diagnosis and prognosis of CAD? |  |
|  | a) X-Ray and Magnetic Resonance Imaging of the chest. |
|  | b) Exercise Treadmill Test (Stress test) and cardiac catheterization (angiogram) |
|  | c) Electrocardiogram (EKG) at rest and a clinical history. |
| 7-Which of the following statements about the management of blood cholesterol levels is most accurate? |  |
|  | a) Physical exercise and diet are enough to lower cholesterol to target levels after a heart attack. |
|  | b) Physical exercise and diet should be followed regularly and when necessary, a medication such as a “statin” may be required |
| c. | ) There is no treatment because high cholesterol levels are genetically inherited and can’t be changed |
| 8-Which of the following statements about the use of “nitroglycerin” is most accurate? |  |
|  | a) They are a class of medications that can be administered to improve coronary blood flow and can be given either continuously (such as in a tablet or patch) or used sublingually (under the tongue as a spray or small tablet) in situations of acute chest pain. |
|  | b) They are medications given only by the sublingual route in emergency situations to relieve chest pain. |
|  | c) They are medicines used to decrease blood pressure and bad cholesterol (LDL) in patients with cardiac problems. |
| 9-Which of the following dietary components best describes a nutritional plan for persons with CAD? |  |
|  | a) A diet with reduced salt, low fat and rich in fiber. |
|  | b) A diet based on whole grains, vegetables, fish, extra virgin olive oil and nuts |
|  | c) An unrestricted diet, because diet is not a relevant factor. |
| 10-Which values for LDL cholesterol and HDL cholesterol are the optimal targets persons with established CAD (values in mmol/litre)? |  |
|  | a) LDL less than 2.0 and HDL greater than 1.2. |
|  | b) LDL 2.0 to 2.5 and HDL greater than 1.0. |
|  | c) LDL greater than 3.0 and HDL less than 1.0 |
| 11-In which of the following situations would you avoid carrying out your regular physical exercise? |  |
|  | a) If you had a recent heart attack (for example 8 weeks ago). |
|  | b) If you have a bad infection today (for example a really bad “flu). |
|  | c) If your blood pressure is moderately elevated (for example 150/90). |
| 12-While walking, if you experience a new episode of severe chest discomfort that you think that is angina, you should: |  |
|  | a) Drive your car directly to the hospital to seek medical care. |
|  | b) Try to relax, wait for the pain to improve, and then seek medical attention |
|  | c) Stop your walk and sit, take a sublingual nitroglycerin, and seek medical care if the pain does not subside. Call your doctor to let him or her know what has happened. |
| 13-Based on your knowledge about exercise and CAD, choose the most appropriate statement below: |  |
|  | a) Physical exercise should never be practiced by patients with coronary artery disease because of high risk of death**.** |
|  | b) Physical exercise is a fundamental part of the treatment plan, because it helps to control risk factors, prolongs survival and enhances quality of life. |
|  | c) Physical activity should be included in the treatment plan only when patients are fully recovered from their heart event. |
| 14-Guidelines for Physical Activity for people with coronary disease should be based upon which of the following: |  |
|  | a) The exercise prescription should be individually devised based on an exercise stress test and respect the person’s abilities and disabilities. |
|  | b) Start at a low level to moderate level and build up gradually. |
|  | c) Be the same for all persons of the same gender and age, because these groups have the same physical ability and risk. |
| 15-Which of the following changes in the body resulting from regular physical exercise are most important to long term cardiac health? |  |
|  | a) Blood vessel function improvement, growth of new blood vessels, and even a possible regression (shrinking) of atherosclerotic plaque. |
|  | b) Resting heart rate decrease, more forceful heart beat, and lipid profile improvement. |
|  | c) Blood Pressure increase, higher heart rates, and higher triglyceride levels. |
| 16-Which of the following statements best describes the pattern for exercise activity in persons recovering from a heart event: |  |
|  | a) At any place, daily duration of about 30 minutes, which can be cumulative (10 min at morning, 10 min at noon and 10 min at night). |
|  | b) In an appropriate setting, with periodic monitoring by qualified professionals, with the goal of achievement of self-sufficiency. |
|  | c) In a hospital environment only. |
| 17-Which of the following statements is the most appropriate guidance around levels of blood pressure levels in persons with CAD: |  |
|  | a) It doesn’t matter whether blood pressure is normal or high because it does not have any long term health effects. |
|  | b) A value of less than 140 / 90 mmHg is considered normal. |
|  | c) An optimal blood pressure is 120 / 80 mmHg. |
| 18-Which of the statements below regarding psychological stress is most correct? |  |
|  | a) It is one of the important risk factors for AMI (Acute Myocardial Infarction). |
|  | b) Stress is related to the presence of anxiety and depression. |
|  | c) It has no impact on heart disease, since atherosclerosis is a completely physical process and is not related to psychological factors. |
| 19-Which interventions can extend and improve a patient’s quality of life for persons recovering from a cardiac event? |  |
|  | a) Lifestyle changes + medical treatments + in some cases surgical intervention. |
|  | b) Medication + in some cases surgical intervention. |
|  | c) Prolonged bed rest. |
