# Supplementary material for: Cultural adoption, and validation of the Persian version of the coronary artery disease education questionnaire (CADE-Q): a second-order confirmatory factor analysis
Source: BMC Cardiovasc Disord. 2020 Jul 23;20:345. doi: 10.1186/s12872-020-01628-5 (PMC7379361; doi:10.1186/s12872-020-01628-5)
Supplement: Supplementary file 2 — Additional file 2. Inter-item correlations for the CADE-Q. [file 12872_2020_1628_MOESM2_ESM.doc]

Inter-item correlations for the CADE-Q

|  | Q1 | Q2 | Q3 | Q4 | Q5 | Q6 | Q7 | Q8 | Q9 | Q10 | Q11 | Q12 | Q13 | Q14 | Q15 | Q16 | Q17 | Q18 | Q19 |
| --- | --- | --- | --- | --- | --- | --- | --- | --- | --- | --- | --- | --- | --- | --- | --- | --- | --- | --- | --- |
| Q1 | 1 |  |  |  |  |  |  |  |  |  |  |  |  |  |  |  |  |  |  |
| Q2 | 0.311^**^ | 1 |  |  |  |  |  |  |  |  |  |  |  |  |  |  |  |  |  |
| Q3 | 0.102 | 0.356^**^ | 1 |  |  |  |  |  |  |  |  |  |  |  |  |  |  |  |  |
| Q4 | 0.377^**^ | 0.236^**^ | 0.190^**^ | 1 |  |  |  |  |  |  |  |  |  |  |  |  |  |  |  |
| Q5 | 0.319^**^ | 0.312^**^ | 0.130^*^ | 0.215^**^ | 1 |  |  |  |  |  |  |  |  |  |  |  |  |  |  |
| Q6 | 0.279^**^ | 0.289^**^ | 0.229^**^ | 0.291^**^ | 0.321^**^ | 1 |  |  |  |  |  |  |  |  |  |  |  |  |  |
| Q7 | 0.356^**^ | 0.324^**^ | 0.181^**^ | 0.325^**^ | 0.246^**^ | 0.399^**^ | 1 |  |  |  |  |  |  |  |  |  |  |  |  |
| Q8 | 0.255^**^ | 0.269^**^ | 0.272^**^ | 0.306^**^ | 0.259^**^ | 0.381^**^ | 0.386^**^ | 1 |  |  |  |  |  |  |  |  |  |  |  |
| Q9 | 0.376^**^ | 0.302^**^ | 0.143^*^ | 0.466^**^ | 0.129^*^ | 0.303^**^ | 0.355^**^ | 0.256^**^ | 1 |  |  |  |  |  |  |  |  |  |  |
| Q10 | 0.311^**^ | 0.262^**^ | 0.154^*^ | 0.145^*^ | 0.240^**^ | 0.197^**^ | 0.301^**^ | 0.192^**^ | 0.126^*^ | 1 |  |  |  |  |  |  |  |  |  |
| Q11 | 0.391^**^ | 0.185^**^ | 0.115 | 0.319^**^ | 0.243^**^ | 0.326^**^ | 0.371^**^ | 0.352^**^ | 0.265^**^ | 0.285^**^ | 1 |  |  |  |  |  |  |  |  |
| Q12 | 0.177^**^ | 0.203^**^ | 0.220^**^ | 0.103 | 0.137^*^ | 0.367^**^ | 0.239^**^ | 0.241^**^ | 0.230^**^ | 0.236^**^ | 0.306^**^ | 1 |  |  |  |  |  |  |  |
| Q13 | 0.269^**^ | 0.199^**^ | 0.138^*^ | 0.412^**^ | 0.172^**^ | 0.247^**^ | 0.220^**^ | 0.287^**^ | 0.288^**^ | 0.105 | 0.385^**^ | 0.217^**^ | 1 |  |  |  |  |  |  |
| Q14 | 0.342^**^ | 0.251^**^ | 0.200^**^ | 0.409^**^ | 0.132^*^ | 0.256^**^ | 0.395^**^ | 0.401^**^ | 0.359^**^ | 0.270^**^ | 0.468^**^ | 0.193^**^ | 0.455^**^ | 1 |  |  |  |  |  |
| Q15 | 0.386^**^ | 0.235^**^ | 0.145^*^ | 0.415^**^ | 0.170^**^ | 0.275^**^ | 0.355^**^ | 0.389^**^ | 0.379^**^ | 0.220^**^ | 0.358^**^ | 0.083 | 0.454^**^ | 0.601^**^ | 1 |  |  |  |  |
| Q16 | 0.286^**^ | 0.259^**^ | 0.144^*^ | 0.319^**^ | 0.232^**^ | 0.250^**^ | 0.320^**^ | 0.400^**^ | 0.254^**^ | 0.276^**^ | 0.377^**^ | 0.138^*^ | 0.369^**^ | 0.492^**^ | 0.576^**^ | 1 |  |  |  |
| Q17 | 0.191^**^ | 0.225^**^ | 0.204^**^ | 0.110 | 0.246^**^ | 0.221^**^ | 0.237^**^ | 0.305^**^ | 0.070 | 0.194^**^ | 0.192^**^ | 0.296^**^ | 0.255^**^ | 0.245^**^ | 0.191^**^ | 0.317^**^ | 1 |  |  |
| Q18 | 0.278^**^ | 0.201^**^ | 0.128^*^ | 0.389^**^ | 0.187^**^ | 0.335^**^ | 0.393^**^ | 0.325^**^ | 0.378^**^ | 0.198^**^ | 0.327^**^ | 0.160^*^ | 0.417^**^ | 0.473^**^ | 0.459^**^ | 0.437^**^ | 0.287^**^ | 1 |  |
| Q19 | 0.229^**^ | 0.232^**^ | 0.089 | 0.476^**^ | 0.105 | 0.398^**^ | 0.421^**^ | 0.328^**^ | 0.382^**^ | 0.194^**^ | 0.327^**^ | 0.169^**^ | 0.478^**^ | 0.512^**^ | 0.551^**^ | 0.475^**^ | 0.240^**^ | 0.623^**^ | 1 |

**. Correlation is significant at the 0.01 level

*. Correlation is significant at the 0.05 level
