# Supplementary material for: Cultural adoption, and validation of the Persian version of the coronary artery disease education questionnaire (CADE-Q): a second-order confirmatory factor analysis
Source: BMC Cardiovasc Disord. 2020 Jul 23;20:345. doi: 10.1186/s12872-020-01628-5 (PMC7379361; doi:10.1186/s12872-020-01628-5)
Supplement: Supplementary file 3 — Additional file 3. The EFA for the CADE-Q among patients with and without hospitalization history. [file 12872_2020_1628_MOESM3_ESM.doc]

**The EFA for the CADE-Q among patients with and without hospitalization history**

|  | **With hospitalization (n = 329)** | **Without hospitalization (n = 171)** |
| --- | --- | --- |
| **Factor** | **Factor loading** | **Factor loading** |
| **Lifestyle habits & exercise** |  |  |
| 19-Which interventions can extend and improve a patient’s quality of life for persons recovering from a cardiac event? | 0.99 | 0.99 |
| 15-Which of the following changes in the body resulting from regular physical exercise are most important to long term cardiac health? | 0.76 | 0.71 |
| 18-Which of the statements below regarding psychological stress is most correct? | 0.45 | 0.43 |
| 14-Guidelines for Physical Activity for people with coronary disease should be based upon which of the following: | 0.85 | 0.78 |
| 13-Based on your knowledge about exercise and CAD, choose the most appropriate statement below: | 0.54 | 0.52 |
| 4- Which of the following statements is most accurate regarding our understanding of CAD? | 0.39 | 0.33 |
| 16-Which of the following statements best describes the pattern for exercise activity in persons recovering from a heart event: | 0.54 | 0.50 |
| *Eigenevalue* | *6.32* | *5.49* |
| *% variance* | *20.00* | *14.06* |
| **Risk factors** |  |  |
| 3-Which description below is a typical symptom of CAD | 0.36 | 0.33 |
| 2-Which factors have the most influence on the risk of myocardial infarction. | 0.44 | 0.42 |
| 6-Of the investigations listed below, which ones provide the most precise information about the diagnosis and prognosis of CAD? | 0.47 | 0.43 |
| 5-The best time of the day for people with coronary disease to carry out their prescribed exercise is: | 0.63 | 0.61 |
| 7-Which of the following statements about the management of blood cholesterol levels is most accurate? | 0.34 | 0.30 |
| *Eigenevalue* | *1.40* | *1.91* |
| *% variance* | *6.60* | *17.61* |
| **Diagnosis and treatment** |  |  |
| 1-Coronary Artery Disease (CAD) is: | 0.34 | 0.32 |
| 17-Which of the following statements is the most appropriate guidance around levels of blood pressure levels in persons with CAD: | 0.99 | 0.95 |
| 10-Which values for LDL cholesterol and HDL cholesterol are the optimal targets persons with established CAD (values in mmol/litre)? | 0.33 | 0.31 |
| 11-In which of the following situations would you avoid carrying out your regular physical exercise? | 0.57 | 0.55 |
| *Eigenevalue* | *1.13* | *1.17.* |
| *% variance* | *11.50* | *7.46* |
| **Signals**  **& symptoms and medicine** |  |  |
| 12-While walking, if you experience a new episode of severe chest discomfort that you think that is angina, you should: | 0.71 | 0.68 |
| 8-Which of the following statements about the use of “nitroglycerin” is most accurate | 0.34 | 0.32 |
| *Eigenevalue* | *1.08* | *1.06* |
| *% variance* | *4.17* | *3.20* |
